# Effectiveness of Bariatric Surgery in the Management of Type 2 Diabetes Mellitus: A Case Report and Literature Review

**DOI:** 10.7759/cureus.47843

**Published:** 2023-10-27

**Authors:** Bashir Mahamud, Rohan K Suthar, Ayan Mohamed, Kedir Hamid, Gideon Mlawa

**Affiliations:** 1 Medicine, Trakia Medical University, Stara Zagora, BGR; 2 Acute Medicine, Barking, Havering and Redbridge University Hospitals NHS Trust, London, GBR; 3 Respiratory Medicine, Barking, Havering and Redbridge University Hospitals NHS Trust, London, GBR; 4 Internal Medicine, Barking, Havering and Redbridge University Hospitals NHS Trust, London, GBR; 5 Internal Medicine and Diabetes and Endocrinology, Barking, Havering and Redbridge University Hospitals NHS Trust, London, GBR

**Keywords:** type 2 diabetes, bariatric surgery/therapeutic use, gdp expenses, nice guidelines, glycated haemoglobin (hba1c)

## Abstract

Type 2 diabetes (T2DM) and obesity represent major global health burdens and economic costs to healthcare systems. T2DM management is challenging due to multiple comorbidities and limited drug efficacy. Bariatric surgery has emerged as an effective treatment approach. A 65-year-old man with refractory obesity (BMI > 35 kg/m^2^) and poorly controlled T2DM underwent gastric bypass surgery in 2018. Prior to surgery, medication noncompliance and dietary measures failed to achieve adequate glycemic control or weight loss. Postoperatively, the patient lost 20 kg and achieved improved T2DM control (HbA1C reduction), allowing complete cessation of diabetic medications. The patient's case demonstrates bariatric surgery's potential to significantly alter the clinical course of obesity and T2DM versus standard care. National guidelines outline eligibility criteria for bariatric referral; however, utilization rates remain low (<1%) despite over two million eligible individuals in the United Kingdom. Improved access could reduce disease burden and healthcare costs from diabetes complications over the long term. This case report provides a real-world example supporting bariatric surgery as an effective intervention for appropriately selected patients with obesity and uncontrolled T2DM, with the potential to improve clinical outcomes and lower costs associated with diabetes management.

## Introduction

Diabetes is a chronic condition, which has become a major public health concern approaching epidemic proportions globally. It is estimated that approximately 18 million people die every year from cardiovascular disease (CVD), for which diabetes and hypertension are major predisposing factors [1-3].

Type 2 diabetes (T2DM) also known as adult-onset diabetes is characterized by hyperglycemia due to progressive impairment of pancreatic β-cell insulin secretion and/or insulin resistance. T2DM is estimated to affect more than 300 million individuals globally [4]. T2DM has a strong association with non-communicable diseases such as obesity and hypertension [5,6]. Moreover, T2DM is also the leading cause of blindness, kidney failure, and non-traumatic lower limb amputations [7].

Effective diabetes management often presents many challenges. Clinicians and patients are often overwhelmed by the need to address comorbid chronic conditions in addition to patients’ diabetes-specific treatment goals [8].

The economic burden posed by diabetes on national healthcare services such as the NHS is quite substantial. The burden of diabetes can be divided into direct and indirect costs. The direct cost burden of diabetes includes diagnostic and monitoring tests, GP consultations, medications, insulin pumps, continuous monitoring devices, hospital admission, and secondary professional referrals. The indirect costs include social care needs, lost productivity through sickness, absences from work, and hindered quality of life. Combined, the direct and indirect costs of T2DM have been estimated to cost the NHS 21.8 billion pounds annually and are predicted only to increase over the next 20 years. As of 2011, the burden of diabetes on the NHS expenditure was estimated to be 10%. Of this 10%, the indirect costs accounted for more than 60%. Specifically, the indirect cost is due to complications arising from long-term disease burdens such as foot ulcers and amputations, cardiovascular disease, end-stage renal disease, and acute admission due to diabetic emergencies [9].

Studies looking into similar statistics in France and Germany have shown similar trends. The cost of 3000 to 15000 euro per patient increases as insulin therapy is initiated, and most of the costs are attributed to complications and comorbidities due to T2DM [10].

Therefore, it has become of paramount importance to find alternative means of treating diabetes with a more robust outcome that not only minimizes the development of associated comorbidities but provides a more cost-effective alternative to current pharmaceutical drugs.

Currently, the management of diabetes is via a multifaceted approach with lifestyle modifications being the first line. Obesity has been closely linked to poor diabetic control, and weight loss has been shown to lead to significant improvements in glycemic control and reduced medication use in patients with T2DM [11]. This weight loss can be attained not only by aggressive lifestyle changes and medical therapy but also with bariatric surgery.

Bariatric surgery is a group of surgical procedures done with the intent of substantial weight loss in individuals with severe obesity. Bariatric surgery can be done via several techniques. The most common variants are gastric banding, Roux-en-Y gastric bypass, sleeve gastrectomy, and biliopancreatic diversion. They exert their effect by a combination of mechanisms including caloric restriction, reduced absorption, and hormonal changes, which alter the mechanics of appetite and satiety [12].

It is widely accepted that bariatric surgery can help improve health outcomes in a range of metabolic diseases, including T2DM and obesity [13]. Bariatric surgeries promote weight loss, which can lead to improvements in hyperglycemic and dyslipidemia control. Some studies have shown that weight loss attained via bariatric surgery is greater and longer lasting when compared to nonsurgical methods [14]. Recent data from a national obesity audit has shown that the use of bariatric surgery has fallen by more than 30 percent in the last four years in the United Kingdom.

Herein, we present a case of a patient with poorly controlled T2DM and obesity who was effectively managed with elective bariatric surgery. Despite the significant drop in the number of bariatric procedures performed in this country, this case highlights that bariatric procedures should be considered as not only a viable treatment option for patients but also a cost-effective means of treating these patients.

This case was presented at the European Endocrine Society at the 22nd European Congress of Endocrinology, which took place from September 5th to 9th, 2020, as an abstract and poster.

## Case presentation

A 65-year-old man was referred to the endocrine team with poor diabetic control, ongoing chronic kidney disease, and previous history of non-alcoholic fatty liver and prostate hyperplasia, for which he was taking Metformin (1g BD), Forxiga (10 mg OD) (dapagliflozin), Finasteride (5 mg OD), and Alfuzosin (10 mg OD). On physical examination, the patient's weight was 131 kg, his heart rate was 99 bpm, and his blood pressure was 154/88 mmHg. The foot examination was unremarkable. There was no evidence of peripheral neuropathy or ulceration. Laboratory findings showed urea of 8.8, creatinine of 114 umol/L, eGFR > 60 ml/min, and hemoglobin A1C (HbA1C) of 77 mmol/mol.

He indicated that he gained weight over the past few months. He also indicated that he felt more fatigued and tired. The patient was referred to a dietician to help with his weight loss and diabetic control. The patient was again seen by the endocrine team six months later, and his weight remained alarmingly high (126 kg) despite consulting a dietician. In addition, his diabetes remained uncontrolled. At this point, he was given an additional antidiabetic drug Victoza (1.2 mg OD) and was referred to Homerton Hospital NHS Trust for consideration of bariatric surgery in the form of a gastric band.

Following the bariatric surgery, he lost a significant amount of weight and showed better glycemic control. His medication following bypass surgery included multivitamins, especially Vitamin B12 and Vitamin D with all previous diabetes medication stopped.

**Figure 1:**
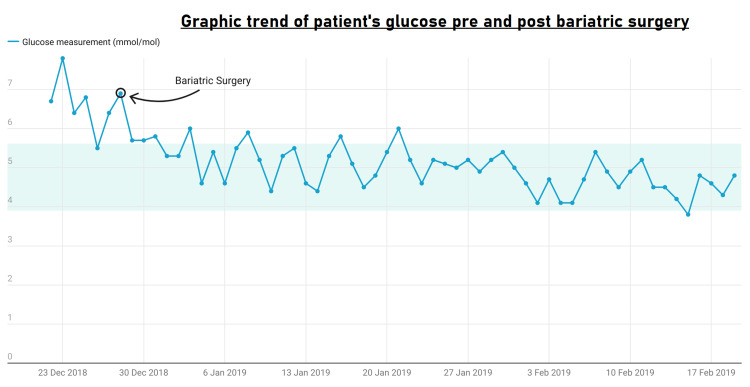
Patient glucose levels after undergoing gastric band bariatric surgery on December 28, 2018

**Figure 2:**
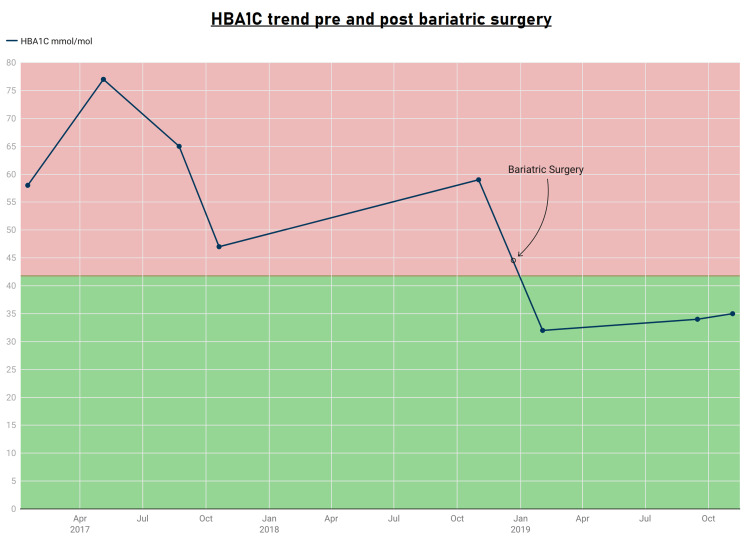
HbA1C results before and after gastric band bariatric surgery on December 28, 2018 HbA1C: Hemoglobin A1C.

The patient's glucose levels after undergoing gastric band bariatric surgery are shown in Figure 1, and Figure 2 shows the HbA1C results before and after gastric band bariatric surgery.

The patient's glucose readings after undergoing gastric band surgery are shown in Table 1, and Table 2 shows the patient's HbA1C before and after gastric band surgery.

**Table 1:** Glucose readings after undergoing gastric band surgery on December 28, 2018

Date	Glucose measurement (mmol/mol)	Date	Glucose measurement (mmol/mol)
22/12/2018	6.7	21/01/2019	6
23/12/2018	7.8	22/01/2019	5.2
24/12/2018	6.4	23/01/2019	4.6
25/12/2018	6.8	24/01/2019	5.2
26/12/2018	5.5	25/01/2019	5.1
27/12/2018	6.4	26/01/2019	5
28/12/2018	6.9	27/01/2019	5.2
29/12/2018	5.7	28/01/2019	4.9
30/12/2018	5.7	29/01/2019	5.2
31/12/2018	5.8	30/01/2019	5.4
01/01/2019	5.3	31/01/2019	5
02/01/2019	5.3	01/02/2019	4.6
03/01/2019	6	02/02/2019	4.1
04/01/2019	4.6	03/02/2019	4.7
05/01/2019	5.4	04/02/2019	4.1
06/01/2019	4.6	05/02/2019	4.1
07/01/2019	5.5	06/02/2019	4.7
08/01/2019	5.9	07/02/2019	5.4
09/01/2019	5.2	08/02/2019	4.9
10/01/2019	4.4	09/02/2019	4.5
11/01/2019	5.3	10/02/2019	4.9
12/01/2019	5.5	11/02/2019	5.2
13/01/2019	4.6	12/02/2019	4.5
14/01/2019	4.4	13/02/2019	4.5
15/01/2019	5.3	14/02/2019	4.2
16/01/2019	5.8	15/02/2019	3.8
17/01/2019	5.1	16/02/2019	4.8
18/01/2019	4.5	17/02/2019	4.6
19/01/2019	4.8	18/02/2019	4.3
20/01/2019	5.4	19/02/2019	4.8

**Table 2:** HbA1C before and after gastric band surgery on December 28, 2018 HbA1C: Hemoglobin A1C.

Date	HbA1C (mmol/mol)
15/01/2017	58
05/05/2017	77
23/08/2017	65
20/10/2017	47
01/11/2018	59
02/02/2019	32
15/09/2019	34
05/11/2019	35

## Discussion

T2DM, obesity, hypertension, and dyslipidemia collectively carry a high risk of cardiovascular disease and mortality. Diabetes is also a leading cause of non-traumatic lower extremity amputation and blindness in the United Kingdom. The majority of diabetes-related amputations in England are preventable if patients get the right early preventative care upon recognition of uncontrolled diabetes.

Medical management of T2DM and obesity is challenging as drug therapies have limited efficacy due to side effects and noncompliance. However, bariatric surgery has shown promise as an effective treatment. This case report details one patient who underwent gastric bypass surgery in December 2018. He lost 20 kg post-surgery and demonstrated significantly improved diabetic control, with glucose levels ranging from 4.5 to 6.0 mmol/L. His HbA1C gradually declined, and he was ultimately able to stop all diabetic medications (Figure 1, Table 1). Additionally, the patient reported improvements in psychological and physical well-being.

Without surgery, this patient likely would have continued struggling with poor glycemic control over time. Many patients have worse control over time, become less compliant with management, and require more medication and hospitalization. Additionally, through countless studies, there is strong evidence that prolonged poor glycemic control is associated with the development of both macro and microvascular complications in a short period of time.

In 2014, the National Institute for Health and Care Excellence (NICE) established guidelines for the use of bariatric surgery. To be eligible, patients required a BMI ≥ 40 kg/m^2^ or 35-40 kg/m^2^ with obesity-related comorbidities that may improve with weight loss. All appropriate nonsurgical weight loss methods also needed to be attempted with minimal success. Additionally, NICE recommended expediting bariatric surgery for patients with BMI ≥35 kg/m^2^ and recent onset T2DM (<10 years), with lower BMI thresholds (reduced by 2.5 kg/m^2^) for Asian, Middle Eastern, Black African, or African-Caribbean family background [15].

Based on these criteria, over two million people in the United Kingdom were estimated to be eligible for bariatric surgery. However, a study found that less than 1% of eligible patients were referred for and received the intervention, highlighting a gap between clinical need and access [16]. More concerningly, bariatric surgery rates have fallen over 30% from 2019 to the present, despite the increasing evidence of effectiveness, demonstrating a clear imbalance between patient demand and available supply [17]. Improving access to bariatric surgery for appropriately selected patients could help address obesity and diabetes more effectively.

Bariatric surgery is well-established as an effective treatment modality for patients with obesity and T2DM. Several studies and case reports have highlighted the key role bariatric surgery can have in achieving both weight loss and T2DM remission [18]. A recent study [19] indicated that 72% of T2DM patients treated with bariatric surgery achieved diabetes remission after two years. Randomized control trials have shown that bariatric surgery consistently outperforms best medical therapy in improving glycemic control leading to remission; however, there appears to be clinical reluctance for its widespread use in treating severe T2DM. Recent research highlighted that in the NHS, bariatric surgery is used for people with T2DM at a much later stage in the disease process [20].

This case suggests that bariatric surgery could significantly alter the course of patients with obesity and uncontrolled diabetes, reducing associated morbidity and mortality. If these findings were extrapolated to a larger population, offering the option of such an intervention to a greater number of individuals could effectively improve patient outcomes and significantly reduce the cost burden on the NHS.

Chronic conditions such as T2DM are causing strain on national healthcare. Since 2010, there has been a one percent increase in healthcare spending in England. The cost of treating diabetes by the NHS has increased annually in connection with increased incidence of T2DM and obesity. Diabetic therapy is currently costing the NHS over one billion [21]. Along with treating diabetes, there is also an added cost of in-home care for diabetic patients as they require glucometer and needles, which are provided to patients to measure their glucose levels.

Studies estimate the cost of providing bariatric surgery for patients with obesity and T2DM can be recouped within three years via reduced prescriptions per patient and less hospitalization [12].

Limitations

This is a case study and thus cannot predict the confounding factors that may occur in a larger population. Additionally, there is only a limited time span that the patient was monitored. Long-term advantages may not be as significant as predicted. The case may lack generalizability to a wider population.

## Conclusions

Bariatric surgery is a robust means of treating diabetes and obesity. The use of this would limit both the overprescription of antidiabetic therapy and its cost, which at the moment is estimated to cost the NHS over a billion pounds. Moreover, it would improve the psychological and physical well-being of patients hindered by T2DM.

## References

[1] American Diabetes Association (2018). 2. Classification and Diagnosis of Diabetes: Standards of Medical Care in Diabetes-2018. Diabetes Care.

[2] Al-Goblan AS, Al-Alfi MA, Khan MZ (2014). Mechanism linking diabetes mellitus and obesity. Diabetes Metab Syndr Obes.

[3] Tabish SA (2007). Is diabetes becoming the biggest epidemic of the twenty-first century?. Int J Health Sci (Qassim).

[4] Rodríguez-Gutiérrez R, Montori VM (2016). Glycemic control for patients with type 2 diabetes mellitus: our evolving faith in the face of evidence. Circ Cardiovasc Qual Outcomes.

[5] Eckel RH, Kahn SE, Ferrannini E (2011). Obesity and type 2 diabetes: what can be unified and what needs to be individualized?. J Clin Endocrinol Metab.

[6] Apovian CM (2016). The obesity epidemic--understanding the disease and the treatment. N Engl J Med.

[7] Pantalone KM, Hobbs TM, Wells BJ (2015). Clinical characteristics, complications, comorbidities and treatment patterns among patients with type 2 diabetes mellitus in a large integrated health system. BMJ Open Diabetes Res Care.

[8] Piette JD, Kerr EA (2006). The impact of comorbid chronic conditions on diabetes care. Diabetes Care.

[9] Hex N, Bartlett C, Wright D, Taylor M, Varley D (2012). Estimating the current and future costs of type 1 and type 2 diabetes in the UK, including direct health costs and indirect societal and productivity costs. Diabet Med.

[10] Stegbauer C, Falivena C, Moreno A (2020). Costs and its drivers for diabetes mellitus type 2 patients in France and Germany: a systematic review of economic studies. BMC Health Serv Res.

[11] Lean ME, Leslie WS, Barnes AC (2018). Primary care-led weight management for remission of type 2 diabetes (DiRECT): an open-label, cluster-randomised trial. Lancet.

[12] Koliaki C, Liatis S, le Roux CW, Kokkinos A (2017). The role of bariatric surgery to treat diabetes: current challenges and perspectives. BMC Endocr Disord.

[13] Kmietowicz Z (2016). Surgery for obese people with diabetes could save the NHS £100 000 a patient, finds study. BMJ.

[14] Gloy VL, Briel M, Bhatt DL (2013). Bariatric surgery versus non-surgical treatment for obesity: a systematic review and meta-analysis of randomised controlled trials. BMJ.

[15] (2023). Obesity: identification, assessment and management. https://www.nice.org.uk/guidance/cg189/chapter/Recommendations.

[16] Desogus D, Menon V, Singhal R, Oyebode O (2019). An examination of who is eligible and who is receiving bariatric surgery in England: secondary analysis of the health survey for England dataset. Obes Surg.

[17] (2023). Bariatric surgical procedures, 2021/22 (provisional)-National Obesity Audit [management information]. https://digital.nhs.uk/data-and-information/publications/statistical/national-obesity-audit/bariatric-surgical-procedures-2021-22-provisional.

[18] Lauren BN, Lim F, Krikhely A, Taveras EM, Woo Baidal JA, Bellows BK, Hur C (2022). Estimated cost-effectiveness of medical therapy, sleeve gastrectomy, and gastric bypass in patients with severe obesity and type 2 diabetes. JAMA Netw Open.

[19] Zhou X, Zeng C (2023). Diabetes remission of bariatric surgery and nonsurgical treatments in type 2 diabetes patients who failure to meet the criteria for surgery: a systematic review and meta-analysis. BMC Endocr Disord.

[20] Currie A, Bolckmans R, Askari A (2023). Bariatric-metabolic surgery for NHS patients with type 2 diabetes in the United Kingdom National Bariatric Surgery Registry. Diabet Med.

[21] McGlone ER, Carey I, Veličković V (2020). Bariatric surgery for patients with type 2 diabetes mellitus requiring insulin: clinical outcome and cost-effectiveness analyses. PLoS Med.

